# Case Report: A left ventricular pseudoaneurysm detected by cardiac magnetic resonance more than 1 year after a percutaneous transluminal coronary intervention

**DOI:** 10.3389/fcvm.2024.1348750

**Published:** 2024-03-21

**Authors:** Yuanyuan Liu, Ge Xu, Funan Shi, Jing Yang, Ruiqiang Gou, Zixian Chen, Liang Cao

**Affiliations:** ^1^ Department of Radiology, The First Hospital of Lanzhou University, Lanzhou, Gansu, China; ^2^ The First Clinical Medical College, Lanzhou University, Lanzhou, Gansu, China; ^3^ Lanzhou University Second Hospital, Lanzhou University, Lanzhou, Gansu, China; ^4^ Intelligent Imaging Medical Engineering Research Center of Gansu Province, Lanzhou, Gansu, China; ^5^ Accurate Image Collaborative Innovation International Science and Technology Cooperation Base of Gansu Province, Lanzhou, Gansu, China; ^6^ Radiological Clinical Medicine Research Center of Gansu Province, Lanzhou, Gansu, China

**Keywords:** pseudoaneurysm, myocardial infarction, cardiac magnetic resonance, CMR (cardiovascular magnetic resonance), PCI—percutaneous coronary intervention

## Abstract

Pseudoaneurysm is a rare but lethal complication of acute myocardial infarction. In this study, we present a unique case of a patient with left ventricular free wall rupture detected by cardiac magnetic resonance more than 1 year after a percutaneous transluminal coronary intervention.

## Introduction

Ventricular aneurysm is a common complication of acute myocardial infarction (AMI), which can be divided into true aneurysm and false aneurysm (pseudoaneurysm) (1). It has been reported that 0.2%–0.3% of patients with AMI suffer from cardiac rupture, which often occurs within a week of the acute phase (2) but rarely in the chronic phase. Pseudoaneurysm has high rupture and mortality risks, especially if undetected, for which treatment differs from that for true aneurysm (3). Therefore, an early diagnosis of pseudoaneurysm is very important.

In the clinic, it is a challenge to distinguish between true and false ventricular aneurysms. Imaging, especially cardiac magnetic resonance (CMR), plays an important role in the diagnosis and differential diagnosis of a ventricular aneurysm.

In this study, we present a unique case of a patient with a pseudoaneurysm, with a left ventricular (LV) free wall rupture detected by CMR more than one year after a percutaneous transluminal coronary intervention (PCI).

## Case presentation

A 56-year-old man who presented with dizziness and palpitation was admitted to our institution. He was diagnosed with acute inferior wall myocardial infarction (MI) more than 1 year before admission. A coronary angiography (CAG) demonstrated an occluded proximal right coronary artery (RCA) and the first diagonal artery proximal (D1p) and a severe stenosis of the left anterior descending artery proximal (LADp) and left circumflex distal (LCXd); a PCI was performed for the RCA, but it resulted in failure. An echocardiography revealed a massive bloody pericardial effusion; after medication was given, the patient was discharged.

Three months after discharge, he was again admitted to the hospital for a PCI. A stent was implanted in the LAD, but a chronic total occlusion of the RCA was still not opened. A repeat echocardiography showed a minimal pericardial effusion. One month later, the pericardial effusion disappeared and the patient was free of any symptoms.

More than 1 year later, the patient was again admitted to the hospital with dizziness and palpitation. A physical examination showed a blood pressure of 133/92 mmHg, an enlarged cardiac boundary, and low blunt cardiac sounds. Laboratory data showed increased levels of N-terminal pro-B-type natriuretic peptide (9,761 pg/ml; normal range: <350 pg/ml) and D-Dimer (772 ng/ml; normal range: <200 μg/L). Troponin, myoglobin, and creatine kinase MB levels were normal.

**Figure 1:**
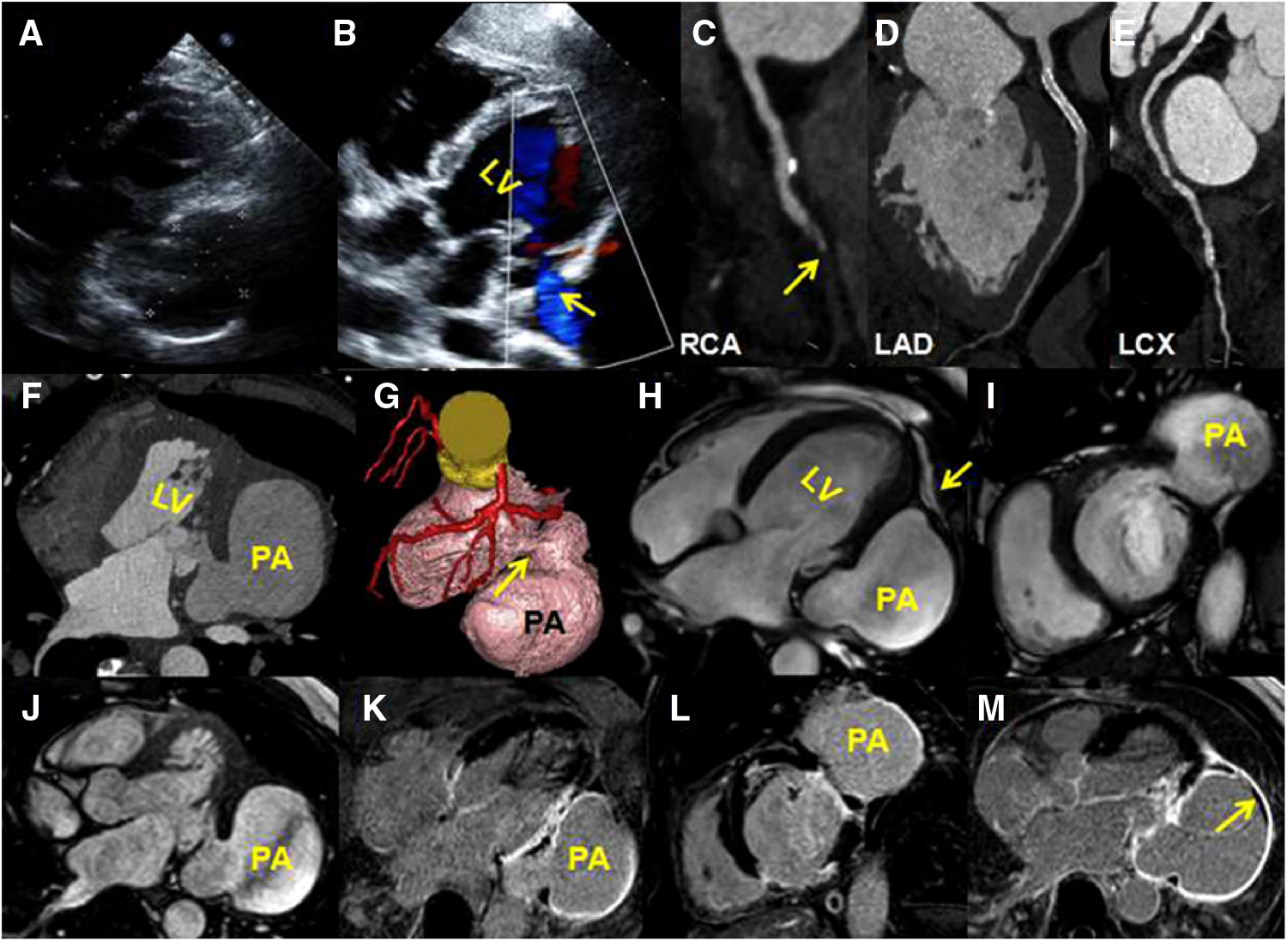
(**A**) A transthoracic four-chamber echocardiography image shows an aneurysm protruding from the posterior wall of the LV; the size of the aneurysm is 6.85 cm × 4.82 cm. (**B**) A color Doppler image shows a shunt from the left ventricle to the aneurysm (yellow arrow). (**C–E**) A coronary computed tomography angiography of curved planar reconstruction images show that the RCA mid-distal is in occlusion (**C**, yellow arrow), the LAD stent is unobstructed (**D**), and there is mild stenosis in the LCX mid-segment (**E**) (**F**) An axial CTA image shows that the lateral wall of the LV is interrupted continuously. (**G**) A volume-rendering CTA image shows a globular cavity and a narrow neck of the aneurysm (yellow arrow). (**H–J**) CMR four-chambered, short-axis and horizontal axis cine images show the aneurysm, and the wall of the aneurysm is continuous with the visceral pericardium (yellow arrow). (**K–M**) Late gadolinium enhancement images show a transmural delay enhancement of the lateral wall of the LV. The enhanced part of the ventricular aneurysm wall is composed of pericardial components. There is a small-sized thrombus around the ventricular pseudoaneurysm (**M**, yellow arrow).

An electrocardiography showed a low ST segment pressure, an increased T wave, and an old inferior wall myocardial infarction. Echocardiography demonstrated that the continuity of theLV lateral wall was interrupted and ventricular aneurysm was formed (6.85 cm × 4.82 cm). Color Doppler showed a shunt blood flow signal from the area of the rupture to the ventricular aneurysm (Figures 1A,B), and the LV ejection fraction (EF) was moderately decreased (41%).

A computed tomography angiography (CTA) showed that the RCA mid-distal was in occlusion (Figure 1C), the LAD stent was unobstructed (Figure 1D), and there was mild stenosis in the LCX mid-segment (Figure 1E). A horizontal axis image showed the ventricular aneurysm of the LV lateral wall (Figure 1F). A volume-rendering image showed that the narrow neck of the LV lateral wall was connected with the ventricular aneurysm (Figure 1G). A CMR was arranged to further clarify the nature of the ventricular aneurysm. The balance steady-state free precession sequence of four-chambered and short-axis images demonstrated that there was a discontinuity of the basal segment of the LV lateral wall, a cystic cavity was seen communicating with the LV (Figures 1H–J, Supplementary Videos S1–S3), and the lateral wall of the cyst was continuous with the pericardium (Figure 1H, yellow arrow). Late gadolinium enhancement (LGE) images showed a transmural myocardial infarction of the LV lateral wall (Figures 1K,L). The wall of the aneurysm was composed of a delayed enhancement of the pericardium rather than the myocardium (Figures 1K,L). A small-sized thrombus could be seen in the aneurysmal wall (Figure 1M, yellow arrow). These signs indicated pseudoaneurysm.

The pseudoventricular aneurysm of the patient was vulnerable to the risk of rupture at any time, and therefore, surgical treatment was recommended. The patient opted for medical management considering his lack of symptoms and the excessive risk involved with surgery.

## Discussion

We identified a LV pseudoaneurysm by CMR in a patient with chronic myocardial infarction more than 1 year after a PCI.

Rupture of ventricular aneurysm is a serious complication after acute MI with high mortality rates. The incidence rate of pseudoaneurysm after MI is in the range of 0.2%–0.3% based on a retrospective study of 1,050 patients by Montrief et al. (4). Cardiac rupture after AMI generally occurs within the first week after its onset; it is the pathological softening stage of MI, and it denotes that both scar repair and fibrosis are not perfect (5). Cardiac rupture can occur under cardiac contraction, diastolic movement, and blood flow impact. The most common rupture site is the anterior and lateral wall of the LV (6, 7). After the rupture of the whole myocardial layer, local thrombosis occurs when the rupture is small, and pericardial tissue and fiber connective tissue wrap the blood to form a cystic cavity called “pseudoaneurysm” (8). The thrombus has a potential risk of systemic embolism, and it reduces the evolution of cardiac rupture during the progression of symptoms in patients. The wall of a pseudoaneurysm is thin, and the ejection capacity of the cardiac rupture increases or decreases, leading to blood flow shock and the rupture of ventricular aneurysm.

In the case of our patient, the LV wall rupture occurred more than 1 year after the PCI, which is a rare event. This chronic rupture has rarely been reported. The incidence rate of LV pseudoaneurysm is low (<1%) and even lower when patients undergo a primary PCI (9). Only a few of these patients pass the critical period and develop LV pseudoaneurysm. Acute tamponade following ventricular wall rupture is also a common cause of death. However, patients with this condition, even after passing the critical period, are still at risk of rupture again due to ischemia, fatigue, irritability, and other factors, with a high risk of mortality (10, 11).

Therefore, early diagnosis and reasonable treatment are crucial in these patients. Only a few patients with pseudoaneurysm present typical symptoms such as congestive cardiac failure, chest pain, and shortness of breath. But most of these are hidden. It has been reported that the location of true aneurysms is different from that of pseudoaneurysm; true aneurysms are posterior or inferior and can therefore be diagnosed according to the location of the lesion. However, a more accurate diagnosis method is based on the different morphological characteristics of aneurysm and pseudoaneurysm in cross-sectional imaging. Echocardiography and CTA are considered good non-invasive imaging modalities for the diagnosis of pseudoaneurysm (12, 13); however, they are not good for determining the nature of the aneurysm. CMR, according to its own characteristics, can be more accurate in determining whether the aneurysm is true or whether it is a pseudoaneurysm.

CMR has the advantages of differentiating an aneurysm from pseudoaneurysm by examining the location of the aneurysm, the neck-to-body ratio of the aneurysm, and the continuity of the myocardial wall (14). For true aneurysms, CMR shows a weakening of the myocardium and extensive scarring at the lesion. The local cardiac function in the infarcted area is significantly weakened, and some patients may have a small amount of pericardial effusion. The method of identification of the thrombus in an aneurysm is better than that of echocardiography. For a pseudoaneurysm, CMR shows that the interruption of ventricular wall continuity and the size of the gap can be measured. A pseudoaneurysm is large and thin, and a thrombus is often present. The neck of the pseudoaneurysm is narrow in size and is surrounded by scar tissue.

Most importantly, unique LGE imaging techniques can determine the composition of the aneurysm wall. The wall of a true aneurysm mainly consists of fibrous scar tissue, which can be seen as a complete delayed enhancement band on CMR, which is continuous with the adjacent normal myocardium without interruption. The wall of a pseudoaneurysm is composed of the pericardium and mural thrombus. CMR shows an irregular delayed enhancement of the aneurysm wall, which interrupts its continuity with the adjacent normal myocardium (15). Although some recent studies also suggest LGE in the pericardium of some pseudoaneurysms and a few true aneurysms, this is quite different from the delayed enhancement of alternative fibrous scar after myocardial infarction (16, 17). Therefore, CMR is the gold standard for diagnosing a pseudoaneurysm.

In most reports it has been revealed that the body of the pseudoaneurysm (epicardium) carries the risk of rupture at any time because of the continuous impact of blood flow from the LV, and acute tamponade following ventricular wall rupture is a common cause of death (18). Long-term prognostic data on the conservative treatment of a left ventricular pseudoaneurysm are limited. Sudden death in most patients is caused by a rupture of acute pseudoaneurysm leading to cardiac tamponade, and therefore, most clinicians recommend surgical repair soon after diagnosis (19, 20). At present, surgery is still the most commonly used treatment. In addition to this, there are medication therapy, percutaneous repair, and cardiac replacement therapy. Studies have shown that in determining the strategy for the internal and surgical treatment of pseudoaneurysms, medications can be administered if the patient's hemodynamic and functional status is stable and there are no complications, in addition to taking into account the patient's wishes (21, 22). Percutaneous repair is a therapeutic method that uses a stiff guidewire inserted in the pseudoaneurysm through the angiographic catheter and then the sheath and dilater can be advanced over this wire. In patients who are not suitable candidates for surgical and transcatheter therapies, including those with significant biventricular failure and with associated end organ impairment, an evaluation for orthotopic heart transplantation may be considered (23, 24).

At present, our patient's condition is stable and asymptomatic. Although the pseudoaneurysm is large, the patient's hemodynamics is stable and the actual stroke volume is sufficient to meet the heart's needs. Therefore, conservative treatment is initiated. It is reported that pseudoaneurysms most often occur after circumflex coronary artery occlusion (25, 26). But in the case of our patient, the LCX is indicated without severe stenosis. Of note, a small intermediate coronary artery is seen in the area supplied by the pseudoaneurysm, and we speculate that it may be involved in the formation of the aneurysm, but the real cause of the rupture still needs further exploration. At present, the patient is convalescing at home and remains in stable condition. He is being followed up at the cardiology clinic and is reported to be doing well.

CMR has unique advantages in terms of judging true and false ventricular aneurysms. Therefore, there is a need to improve the understanding of CMR signs of pseudoaneurysm so as to provide accurate information and make early diagnosis for initiating clinical treatment.

## Data Availability

The original contributions presented in the study are included in the article/Supplementary Material, and further inquiries can be directed to the corresponding authors.
